# Vegetable Grafting: The Implications of a Growing Agronomic Imperative for Vegetable Fruit Quality and Nutritive Value

**DOI:** 10.3389/fpls.2017.00741

**Published:** 2017-05-12

**Authors:** Marios C. Kyriacou, Youssef Rouphael, Giuseppe Colla, Rita Zrenner, Dietmar Schwarz

**Affiliations:** ^1^Department of Vegetable Crops, Agricultural Research InstituteNicosia, Cyprus; ^2^Department of Agricultural Sciences, University of Naples Federico IINaples, Italy; ^3^Department of Agricultural and Forestry Sciences, University of TusciaViterbo, Italy; ^4^Leibniz Institute of Vegetable and Ornamental CropsGroßbeeren, Germany

**Keywords:** carotenoids, Cucurbitaceae, flavor, functional compounds, physiological mechanism, mRNA transport, rootstock, Solanaceae

## Abstract

Grafting has become an imperative for intensive vegetable production since chlorofluorocarbon-based soil fumigants were banned from use on grounds of environmental protection. Compelled by this development, research into rootstock–scion interaction has broadened the potential applications of grafting in the vegetable industry beyond aspects of soil phytopathology. Grafting has been increasingly tapped for cultivation under adverse environs posing abiotic and biotic stresses to vegetable crops, thus enabling expansion of commercial production onto otherwise under-exploited land. Vigorous rootstocks have been employed not only in the open field but also under protected cultivation where increase in productivity improves distribution of infrastructural and energy costs. Applications of grafting have expanded mainly in two families: the Cucurbitaceae and the Solanaceae, both of which comprise major vegetable crops. As the main drives behind the expansion of vegetable grafting have been the resistance to soilborne pathogens, tolerance to abiotic stresses and increase in yields, rootstock selection and breeding have accordingly conformed to the prevailing demand for improving productivity, arguably at the expense of fruit quality. It is, however, compelling to assess the qualitative implications of this growing agronomic practice for human nutrition. Problems of impaired vegetable fruit quality have not infrequently been associated with the practice of grafting. Accordingly, the aim of the current review is to reassess how the practice of grafting and the prevalence of particular types of commercial rootstocks influence vegetable fruit quality and, partly, storability. Physical, sensorial and bioactive aspects of quality are examined with respect to grafting for watermelon, melon, cucumber, tomato, eggplant, and pepper. The physiological mechanisms at play which mediate rootstock effects on scion performance are discussed in interpreting the implications of grafting for the configuration of vegetable fruit physicochemical quality and nutritive value.

## Introduction

Retail cost for fresh horticultural products reflects capital investment in developing suitable plant stock, in fostering its cultivation, and in product storage and handling along the food supply chain. Although a multifaceted concept drawing on various implicated stakeholders, quality is ultimately what captures the expectations to be met at the retail customer’s end of the agroindustry spectrum. The perception of quality is dependent on intrinsic traits of horticultural commodities, shaped by genotypic, cultural and ecophysiological effects, and on extrinsic traits formulated by the socio-economic and marketing environment (Schreiner et al., 2013). Multidisciplinary studies have highlighted that quality is more important to consumers than price when the latter varies within the anticipated range (Harker et al., 2003). Although consonance of quality with the cost of purchase influences consumer behavior, quality is that which largely commands recurring customers. In regulatory context, the issue of quality is addressed chiefly by crop-specific class standards based on limited key visual and organoleptic characteristics (Commission Implementing Regulation (EU) No 543/2011, 2011). Quality standards thus tend to define class criteria for minimum acceptability and provide practical, effective, mostly non-destructive means for standardization procedures. They fail, however, to address complex compositional aspects of quality pertaining to flavor, particularly the volatile aroma fraction, or to nutritional and bioactive value which consumers are becoming increasingly conscious of Schreiner et al. (2013).

Plant breeding on the other hand, has aimed preeminently at improving yield, endowing plant stock with disease resistance, at providing resilience to mechanical injury and improving overall postharvest performance, and to a lesser extent at improving sensory quality traits (Bai and Lindhout, 2007). However, the configuration of important sensory traits, such as volatile aroma components, seems mediated by ethylene-dependent biosynthetic pathways linked also to shelf-life performance (Pech et al., 2008), and to textural changes associated with cell wall matrix solubilization events (Dos-Santos et al., 2013). Hence breeding for shelf-life may elicit adverse pleiotropic effects on desirable sensory attributes (Causse et al., 2002). This is particularly critical in fruits characterized by autocatalytic climacteric ripening, as aptly exemplified by the distinct sensory profile of odorous climacteric vs. inodorous non-climacteric melons (Verzera et al., 2011). Collecting desirable traits while avoiding undesirable combinatorial effects complicates breeding efforts. In this respect, grafting may provide expedient means of selecting independently for rootstock and scion traits, provided the compatibility of the graft combination.

Driven initially by its efficiency as an alternative to the banned use of chlorofluorocarbon-based soil fumigants, the grafting of annual fruit crops has grown across crops and beyond applications restricted to addressing soilborne disease problems (Rouphael et al., 2010). Grafting has been increasingly tapped for cultivation under adverse environments posing abiotic and biotic stresses to vegetable crops, thus enabling expansion of commercial production onto otherwise under-exploited land. The ability of taping wild genetic resources for exploiting traits of root physiological tolerance to stress independently to scion characteristics has facilitated the application of grafting for the cultivation of annual fruit crops under marginal conditions of salinity, nutrient stress, water stress, organic pollutants, and alkalinity (Savvas et al., 2010; Schwarz et al., 2010; Borgognone et al., 2013). Moreover, the economic implications of the significant yield increase imparted by select vigorous commercial rootstocks has encouraged their use under protected cultivation where increase in productivity improves distribution of infrastructural and energy costs (Colla et al., 2011). Provided the anatomical and physiological compatibility of the graft combinations, rootstock effects on plant performance under soil biotic and abiotic stress conditions clearly outweigh those of the scion. Moreover, there is evidence of rootstock mediation in the configuration of scion fruit quality characteristics, widely reported in a range of crops but confounded with frequent rootstock–scion interaction which cannot always be explained in the context of narrow rootstock–scion specificity (Rouphael et al., 2010).

As the main drives behind the expansion of vegetable grafting have been the resistance to soilborne pathogens (Louws et al., 2010), tolerance to abiotic stresses (Schwarz et al., 2010; Kumar et al., 2015; Rouphael et al., 2016) and increase in yields (Lee et al., 2010), rootstock selection and breeding have accordingly conformed to the prevailing demand for improving productivity, arguably at the expense of fruit quality. Thus, grafting has not been employed as a method for improving vegetable fruit quality. Quite the contrary, often yield and quality are contradictory traits (Klee and Tieman, 2013). Considering the rapid expansion of the vegetable grafting industry, understanding the implications of grafting for fruit quality is imperative, and equally pressing is the unraveling of the mechanisms involved. Possible factors engaged in rootstock mediation of quality include changes in water and nutrient uptake efficiency, indirect effects on ripening behavior resulting from altered crop load and source–sink balance, and even an epigenetic component to the grafting process involving transfer of genetic material from rootstock to scion (Savvas et al., 2010; Soteriou et al., 2014; Avramidou et al., 2015). Previous reviews on the grafting of annual fruit crops that have covered aspects relating to fruit quality were published by Davis et al. (2008a, only Cucurbitaceae), Flores et al. (2010 only tomato), and Rouphael et al. (2010). The current review aims at providing an updated critical review of scientific advances addressing grafting effects on the fruit quality of annual crops; moreover, it discusses methodological postulates and mechanisms possibly mediating these effects. Current knowledge has been compiled in a crop specific approach where fruit quality attributes and rootstocks employed are discussed in a uniform and integrated context.

## The Configuration of Fruit Quality in Grafted Vegetables

### Cucurbitaceae

#### Watermelon [*Citrullus lanatus* (Thunb.) Matsum. and Nakai]

The adoption of grafting as a means to secure watermelon crop stand and productivity, mainly against conditions of biotic stress, by far exceeds that of any other open cultivated annual fruit crop (FAO, 2012). The use of rootstocks resistant to soilborne diseases has become a prerequisite for watermelon production, especially in areas where intensive cultivation is practiced and scarcity of arable land precludes the application of broad rotation schemes. While most studies assessing rootstock–scion interaction had initially laid emphasis on aspects of disease resistance and agronomic performance, a plethora of works has been produced that examine the implications of grafting for watermelon fruit quality, involving mainly inter-specific hybrids [*Cucurbita maxima* (Duchesne) × *C. moschata* (Duchesne ex Poir)] and gourd [*Lagenaria siceraria* (Molina) Standl.] rootstocks.

##### Morphometric characteristics

Although a trait prominently delineated by genotype, watermelon fruit weight might be influenced by environmental conditions and cultural practices, including grafting, that affect overall field performance (Alexopoulos et al., 2007; Cushman and Huan, 2008; Proietti et al., 2008; Soteriou and Kyriacou, 2014). Vigorous interspecific and *L. siceraria* rootstocks can improve yields significantly, which in genotypically large-fruited scions usually translates into a tendency for higher unit fruit weight, while in small-fruited cultivars it tends to increase the number of fruits per plant (Colla et al., 2006a; Alexopoulos et al., 2007; Cushman and Huan, 2008; Proietti et al., 2008; Soteriou and Kyriacou, 2014). Decrease in fruit weight against non-grafted control is usually an indicator of rootstock–scion incompatibility, while in compatible grafts maximum reported fruit weight increase approximates 55% (Yetisir and Sari, 2003; Yetisir et al., 2003; Huitrón et al., 2007; Cushman and Huan, 2008; Soteriou and Kyriacou, 2014).

Secondary morphological characteristics of watermelon fruit that may appeal to consumers’ perception of quality include shape, expressed as the ratio of longitudinal to equatorial diameter, and rind thickness. Fruit shape constitutes a trait predominantly governed by scion genotype and little affected by environmental or cultural factors; hence the effect of grafting thereupon has been circumstantial and mostly non-significant or minimal (Colla et al., 2006a; Alan et al., 2007; Rouphael et al., 2008; Soteriou and Kyriacou, 2014; Fredes et al., 2017). On the other hand, rind thickness is a morphological trait more responsive to grafting, and to cultural practice at large, as it relates to watermelon harvest maturity (Soteriou et al., 2014; Kyriacou et al., 2016). On commercial *C. maxima* × *C. moschata* and *L. siceraria* rootstocks, especially on landraces of the latter, thickening of watermelon rind is often observed (Yetisir et al., 2003; Alexopoulos et al., 2007; Proietti et al., 2008; Kyriacou and Soteriou, 2015). However, this has not been a ubiquitous effect across the above rootstocks, or with less common rootstocks such as *C. moschata*, *Sicyos angulatus* L., *C. lanatus* var. *citroides* (L. H. Bailey) Mansf. and *C. pepo* L. which were only sporadically effective in this respect (Davis and Perkins-Veazie, 2005; Alan et al., 2007; Huitrón et al., 2008; Soteriou and Kyriacou, 2014; Fredes et al., 2017). Rootstock effect on watermelon rind thickness is in general limited and studies involving multiple rootstock–scion combinations have demonstrated the predominance of the relative effect of the scion cultivar on this attribute (Kyriacou and Soteriou, 2015). Thinning of the rind is known to characterize watermelon maturation, but also its postharvest life (Corey and Schlimme, 1988); therefore, potential rootstock effect should be examined under conditions that account carefully for the effect of harvest maturity (Soteriou et al., 2014). In any case, thickening of the rind can improve the postharvest performance of watermelon fruit and may also provide a tool for increasing the source of important bioactive compounds concentrated in the rind, such as citrulline, which constitute potential by-products of the fresh-cut industry (Tarazona-Díaz et al., 2011).

##### Colourimetric attributes

Among the physical characteristics of watermelon fruit that strongly influence consumer preference, is the intensity of red coloration of the pulp. Change in the intensity of red hue, expressed as increase in colourimetric CIELAB component a^∗^, marks the development of watermelon pulp color during ripening; moreover, a widening of hue angle (h°), signifying transition from red to orange–yellow is characteristic of watermelon over-ripening and senescence (Brown and Summers, 1985; López–Galarza et al., 2004; Soteriou et al., 2014). Watermelon pulp color is directly dependent upon lycopene synthesis and its accumulation in chromoplasts, while cultivar differences in pulp color correlate highly with differences in lycopene content (Perkins-Veazie and Collins, 2006; Kyriacou and Soteriou, 2015). Grafting therefore may affect pulp color to the extent it affects lycopene content (Davis and Perkins-Veazie, 2005). Watermelon scions grafted on interspecific *Cucurbita* hybrid rootstocks may incur delayed pulp color development, compared to non-grafted control, expressed as a delayed peak in colourimetric component a^∗^ synchronous to the peak in lycopene content (Soteriou et al., 2014).

##### Textural characteristics

Pulp firmness constitutes one of the most important sensory traits of watermelon fruit subject to wide genotypic variation, with pronounced firmness observed usually in seedless, triploid cultivars (Leskovar et al., 2004; Soteriou and Kyriacou, 2015). Notwithstanding the genotypic effect of the scion, rootstock effects on watermelon pulp firmness can be significant hence the choice of rootstock instrumental for improving fruit quality and postharvest life (Yetisir et al., 2003; Cushman and Huan, 2008; Bruton et al., 2009; Kyriacou and Soteriou, 2015). Inter-specific *Cucurbita* hybrid rootstocks most consistently increase watermelon pulp firmness in both diploid and triploid scions (Bruton et al., 2009; Huitrón et al., 2009; Soteriou et al., 2014; Soteriou and Kyriacou, 2015). The effect of grafting, however, might render the pulp of certain cultivars, especially mini triploids that are genotypically inclined to outstanding firmness, undesirably hard (Soteriou and Kyriacou, 2015). Among less commonly used rootstocks, the parents of interspecific hybrids *C. maxima* and *C. moschata*, *C. ficifolia* Bouché, and citron melon (*C. lanatus* var. *citroides*) have been reported to elicit firmer watermelon pulp (Cushman and Huan, 2008; Bruton et al., 2009), whereas cushaw squash (*C. argyrosperma* C. Huber) pumpkin had the opposite effect (Davis and Perkins-Veazie, 2005). Gourd rootstocks *L. siceraria* usually have no effect on pulp firmness although erratic cultivar-specific effects, both positive and negative, have been reported (Yetisir et al., 2003; Cushman and Huan, 2008; Bruton et al., 2009; Özdemir et al., 2016). Morphological abnormalities scarcely associated with watermelon grafting include yellow bands in the pulp bordering the rind, hollow heart, excessively hard and discolored pith, and overall poor texture (Lee, 1994; Yamasaki et al., 1994; Davis et al., 2008b; Soteriou and Kyriacou, 2014). However, most reports on commercially available *C. maxima* × *C. moschata* and *L. siceraria* rootstocks do not make reference to such defects which may reflect rootstock–scion incompatibility and adverse environmental conditions or cultural practices.

##### Sweetness and acidity

The most valued singular quality trait of watermelon is undoubtedly sweetness, sensorially triggered mostly but not entirely by soluble mono- and di-saccharides, since other juice solutes including organic acids, soluble pectins and amino acids, phenolic compounds and minerals influence sweet sensation (Kader, 2008; Magwaza and Opara, 2015). The soluble solids content (SSC) – containing sugars and acids, together with small amounts of dissolved vitamins, fructans, proteins, pigments, phenolics, and minerals – is the most important quality measure used to indicate sweetness of watermelon as well as other fruits (Magwaza and Opara, 2015). It is in general not highly compromised by grafting on most commercial *C. maxima* × *C. moschata* rootstocks (Colla et al., 2006a; Proietti et al., 2008; Huitrón et al., 2009; Soteriou and Kyriacou, 2014; Kyriacou et al., 2016). Scion response to *L. siceraria* rootstocks appears more erratic and rootstock-specific with most graft combinations not demonstrating a significant effect on SSC but exceptions of SSC reduction, especially on landraces, or SSC increase are not infrequent (Yetisir and Sari, 2003; Alan et al., 2007; Alexopoulos et al., 2007; Cushman and Huan, 2008; Çandır et al., 2013). Effects on watermelon sweetness have occasionally been demonstrated by more marginal or experimental rootstocks, such as reduction of SSC by *C. argyrosperma* and *C. pepo* (Davis and Perkins-Veazie, 2005), and increase by *C. lanatus* var. *citroides* (Fredes et al., 2017).

Sweetness depends mostly on the total concentration of soluble carbohydrates, which in most fruits constitutes the largest fraction of the SSC, but also on the relative proportions of the three main sugars, glucose, fructose, and sucrose, which contribute differentially to sweetness and combine to yield what is termed sweetness index (Elmstrom and Davis, 1981; Brown and Summers, 1985; Kader, 2008). Among cucurbit genotypes, variation in sugar content and sweetness index has been associated mostly with their ability to accumulate sucrose at the expense of fructose and glucose during ripening, owing to the activity of sucrose phosphate synthase and sucrose synthase and the decline in activity of soluble acid invertase (Stepansky et al., 1999; Yativ et al., 2010). In watermelon, fructose and glucose are the main sugars supplying the demands of the ovary during initial fruit development, due to the high activities of neutral and acid invertases (Lanchun et al., 2010). Sucrose is the main soluble carbohydrate accumulating in watermelon fruit during ripening at the expense of reducing sugars (Brown and Summers, 1985; Chisholm and Picha, 1986; López–Galarza et al., 2004; Soteriou et al., 2014), although less common genotypes accumulating reducing-sugars throughout ripening have been reported (Yativ et al., 2010). Lower accumulation of hexoses at the onset of fruit development and reduced sucrose accumulation during ripening have been implicated in moderate reduction of watermelon total sugar content in response to the use of *C. maxima* × *C. moschata* and *L. siceraria* rootstocks (Miguel et al., 2004; Liu et al., 2006; Kyriacou and Soteriou, 2015; Fredes et al., 2017). However, other studies involving the same rootstock types have revealed no significant effects on glucose, fructose, sucrose, or total sugars content (Colla et al., 2006a; Proietti et al., 2008; Soteriou et al., 2014). Disparity of results regarding the effect of grafting on non-structural carbohydrates in many cases reflects differential ripening events, notwithstanding the possible effects of cultural practice and climatic conditions particularly on flowering and fruit setting. Grafting may affect the earliness of flowering and thereby affect the time to commercial maturity (Satoh, 1996; Sakata et al., 2007), however, the delay in maturation relates mainly to retarded post-anthesis ripening events as a result of increased crop load on grafted plants (Soteriou et al., 2014; Kyriacou and Soteriou, 2015; Soteriou and Kyriacou, 2015).

Acidity balances sweetness in the taste profile of most fruits, although effectively the ratio between SSC and titratable acidity (TA) is considered crucial in terms of consumer acceptability mostly for sour fruits. Acidity in watermelon fruit is very low, with a pH range of 5.5–5.8 and acid concentration in its juice 0.7–1.2 g/l predominantly in malate form (Kyriacou and Soteriou, 2012; Çandır et al., 2013; Soteriou et al., 2014; Fredes et al., 2017). Grafting on *C. maxima* × *C. moschata* has been found to increase the TA and reduce the pH of the pulp (Colla et al., 2006a; Proietti et al., 2008; Soteriou et al., 2014). Increase in watermelon acidity has been elicited by grafting not only on hybrid rootstocks but also on *C. lanatus* var. *citroides* and on certain *L. siceraria* rootstocks, expressed mostly in higher malic acid levels in the juice (Çandır et al., 2013; Fredes et al., 2017). The TA of watermelon pulp declines linearly with ripening; grafting, however, sustains higher TA throughout the ripening period in comparison to non-grafted plants, which verifies that this is less mediated by maturity than the effect of grafting on sugars (Soteriou et al., 2014), moreover it predisposes the fruit for improved postharvest performance (Kyriacou and Soteriou, 2015).

##### Aroma profile

Alcohol and aldehyde characteristics of the Cucurbitaceae family constitute the main aroma volatiles in watermelon fruit, with the former usually in higher concentrations (Beaulieu and Lea, 2006; Saftner et al., 2007). The most abundant alcohols identified in the aroma profile of mini watermelons include (*Z*)-3-Nonen-1-ol (fresh melon), (*Z,Z*)-3,6-Nonadien-1-ol (pumpkin, cucumber), hexanol (flower, green), nonanol (herbaceous) and (*Z*)-6-Nonen-1-ol (pumpkin-like, green melon) (Yajima et al., 1985; Dima et al., 2014). Among identified aldehydes most abundant were (*Z*)-2-nonenal (honeydew melon, fruity), hexanal (green), (*E,Z*)-2,6-nonadienal (cucumber, green), nonanal (melon, orange peel), (*Z*)-6-nonenal (honeydew melon, fruity), 6-methyl-5-hepten-2-one (flower) and (*E*)-6-nonenal (earthy) (Dima et al., 2014). Although significant rootstock-specific effects on watermelon volatile profile have been identified, the effect of grafting on watermelon aroma profile remains at large a scarcely charted territory (Petropoulos et al., 2014; Fredes et al., 2017). Grafting midi-watermelon cultivars (≈6 kg) on *C. maxima* × *C. moschata* and *L. siceraria* rootstocks was found to increase fruit content in several aroma volatiles, including (*E*)-2-nonenal (fat, cucumber) and (*Z,Z*)-3,6-nonadien-1-ol (green, cucumber) (Petropoulos et al., 2014). Fredes et al. (2017) identified differential effects among *C. maxima* × *C. moschata* rootstocks in the levels of (*Z*)-6-nonenal and (*E,Z*)-2,6-nonadienal, associated with melon-like and cucumber-like aromas, respectively. A critical and consistent finding across *C. maxima* × *C. moschata* rootstocks, but not on *C. lanatus* var. *citroides*, is the increased level of (*Z*)-6-nonen-1-ol, which confers undesirable pumpkin-like odor in fruits from grafted plants. However, the identification of higher levels of lycopene degradation products, such as 6-methy-5-heten-2-one and geranylacetone in the volatile profile of fruit from non-grafted plants, characterized by earlier peak in lycopene content (Soteriou et al., 2014), suggests differential harvest maturity between treatments may be implicated in these findings (Lewinsohn et al., 2005). Available work is far from providing conclusive evidence on the effect of grafting on watermelon aroma profile. Future work needs to take carefully into consideration the evolution of aroma profile during ripening so that the potential effects of grafting are discerned from those of harvest maturity. Analysis of volatiles performed using a GC–MS-olfactory approach combined with extensively trained sensory panels would provide a more resilient basis for further investigation into rootstock-mediated effects on watermelon aroma profile (Saftner et al., 2007).

##### Functional compounds

Notwithstanding wide genotypic variation, watermelon is a lycopene-rich food source with higher lycopene concentration in its pulp than that of tomato (Perkins-Veazie et al., 2001; Fish and Davis, 2003; Soteriou et al., 2014). Grafting, particularly on *C. maxima* × *C. moschata* rootstocks, has been reported to raise lycopene levels significantly in watermelon fruit (Perkins-Veazie et al., 2007; Proietti et al., 2008; Soteriou et al., 2014; Kyriacou and Soteriou, 2015). Increase was also reported on selected *L. siceraria* genotypes (Çandır et al., 2013) and on *C. argyrosperma* and *C. pepo* but limited to seedless scions (Davis and Perkins-Veazie, 2005). Decrease in lycopene levels associated with certain rootstock–scion combinations involving *L. siceraria* and *C. argyrosperma* (Davis and Perkins-Veazie, 2005; Çandır et al., 2013), or absence of effect (Bruton et al., 2009; Soteriou and Kyriacou, 2014) have been more infrequently reported. Conflicting reports may be explained in the light of recent work demonstrating that lycopene content is affected more by maturity and less by grafting, as the peak in lycopene content appears about 1 week earlier in fruit from non-grafted than from grafted plants (Soteriou et al., 2014). Ripening-dependent accumulation of lycopene may derive from the inhibition of β-carotene synthesis or from an alternative ripening-specific pathway, such as the 1-deoxy-D-xylulose-5-phosphate (DOXP) pathway (Bramley, 2002; Schofield et al., 2008). It is also not known whether the progressive transition in pulp color from red to orange–yellow, which signifies over-ripening, derives from the conversion of accumulated lycopene to β-carotene, or from a senescence-related degradation of lycopene (Ronen et al., 2000; Schofield et al., 2008). The implications of grafting for both of the above processes remain uninvestigated. In addition, lycopene synthesis events are carried over to the postharvest period where they appear temperature-controlled and linked to changes in pulp color (Perkins-Veazie and Collins, 2006). Lycopene content peaked 7 days postharvest at 25°C and was further increased by grafting on *C. maxima* × *C. moschata* rootstocks (Kyriacou and Soteriou, 2015). Depending upon maturity at the time of harvest, postharvest lycopene synthesis may appear as a continuation of the ripening-dependent pattern observed preharvest.

A non-essential amino acid found in abundance in watermelon and other cucurbits is citrulline (Rimando and Perkins-Veazie, 2005). It is a metabolic intermediate in the nitric oxide cycle, active in biological functions such as vasodilation and muscle relaxation which derive from the dissipation of NO during conversion of citrulline to arginine (Nissinen et al., 2003). Earlier indications that grafting could increase amino acid content of watermelon fruit, particularly citrulline (Davis et al., 2008c) have been confirmed by more recent work. Grafting onto *C. maxima* × *C. moschata* rootstock resulted in higher citrulline content in the pulp throughout fruit ripening (Soteriou et al., 2014). Grafting improves the performance of watermelon under deficit irrigation (Proietti et al., 2008), while the accumulation of citrulline in watermelon vegetative tissues under drought conditions has been proposed to contribute to oxidative stress tolerance based on its novel hydroxyl radical scavenging activity (Akashi et al., 2001). Citrulline accumulation in watermelon rind and pulp, possibly relates to an osmotic role during cell expansion as it constitutes a potentially significant fraction of the non-carbohydrate soluble solids in the fruit (Curis et al., 2005; Davis et al., 2011; Tarazona-Díaz et al., 2011; Soteriou et al., 2014).

#### Melon (*Cucumis melo* L.)

Melon constitutes an annual fruit species of complex quality configuration owing to the diverse ripening patterns and associated aroma profiles of its botanical varieties. These are discerned into two major groups: the climacteric short shelf-life odorous varieties *cantalupensis* and *reticulates* (e.g., charentais and muskmelon) characterized by intense aroma, as opposed to the non-climacteric, long shelf-life, non-aromatic *inodorus* varieties, such as honeydew and canary melons (Pech et al., 2008; Allwood et al., 2014). Cantaloupes are among the most widely produced melon varieties but the range of specialty melon types cultivated commercially includes many others, such as Galia, Ananas, Persian, Honeydew, Piel de Sapo, Casaba, Crenshaw, Canary, and Asian melons (Strang et al., 2007). Melon grafting as a phytoprotective measure targets Fusarium and Monosporascus wilts by exploiting mainly resistant same-species (*C. melo*) genotypes, interspecific (*C. maxima* × *C. moschata*) pumpkin hybrids and white gourd [*Benincasa hispida* (Thunb.) Cogn.]; whereas grafting on resistant *Cucumis metuliferus* E. Mey. ex Naudin and *C. melo* subsp. *Agrestis* (Naudin) Pangalo rootstocks emerges as a growing practice against Meloidogyne root knot nematodes (Trionfetti-Nisini et al., 2002; Fita et al., 2007; Davis et al., 2008a; Louws et al., 2010; Guan et al., 2014). Graft incompatibility and deterioration in the fruit quality of grafted plants are common problems, particularly with *Cucurbita* hybrid rootstocks, further complicated by pronounced rootstock interaction with the wide range of melon scion genotypes (Traka-Mavrona et al., 2000; Rouphael et al., 2010; Soteriou et al., 2016).

##### Morphometric characteristics

Whereas compatible *C. melo* and *Cucurbita* hybrid rootstocks generally tend to have no effect on melon fruit weight, there is also widespread rootstock–scion interaction in the responses of different melon types to grafting. For instance, the fruit weight of muskmelon (cv. Proteo) was not influenced by either *C. melo* (cvs. Energia and Sting) or *Cucurbita* hybrid rootstocks (cvs. Polifemo, AS10, RS841, P360, and Elsi) (Condurso et al., 2012). However, the same scion (cv. Proteo) grown hydroponically on other *C. melo* (cvs. Dinero and Jador) and hybrid rootstocks (cvs. P360 and PS1313), incurred a limited mean increase of 6.8% in fruit weight (Colla et al., 2010a). In the case of inodorus honeydew melon (cv. Incas), fruit weight was not influenced by grafting onto *C. melo* (cvs. Belimo, Energia, Griffin, Sting, and ES liscio) and *Cucurbita* hybrid rootstocks (cvs. AS10, P360, ES99-13, and Elsi), although it was increased moderately when grafted onto hybrids ‘RS841’ and ‘Polifemo’ (Crinò et al., 2007; Verzera et al., 2014). Commercial hybrid rootstocks ‘TZ148,’ ‘N101,’ ‘Carnivor,’ and ‘30900’ also had no effect on the fruit weight of an Ananas type (cv. Raymond) and two Galia type (cvs. Elario and Polynica) melons (Soteriou et al., 2016), as was also the case with cantaloupe (cv. Athena) grafted on interspecific hybrids ‘Strong Tosa’ and ‘Tetsukabuto’ (Zhao et al., 2011). By contrast, Schultheis et al. (2015) identified a general trend for reduction of fruit weight as a result of grafting in field trials of muskmelon, honeydew and specialty melons, tested, however, only on hybrid rootstock cv. Carnivor. Traka-Mavrona et al. (2000) found grafting had no effect on fruit weight of three inodorous melons (cvs. Thraki, Peplo, and Lefko Amynteou), and a cantaloupe (cv. Kokkini Banana) using two hybrids (‘TZ-148’ and ‘Mamouth’) and one pumpkin (*C. maxima*) landrace (‘Kalkabaki’) as rootstocks under protected and open field cultivation. Similarly, grafting galia (cv. Arava) and honeydew (cv. Honey Yellow) melons onto nematode-resistant *C. metuliferus* had no effect on fruit weight under organic or conventional production systems (Guan et al., 2014). Exceptional increase (29%) in fruit weight was reported for cantaloupe (cv. Cyrano) when grafted on hybrid ‘P360’ and grown under greenhouse salinity treatments (Colla et al., 2006b). Decrease in fruit weight of muskmelon cv. Proteo resulted from grafting onto *B. hispida*, whereas *C. metuliferus*, *C. zeyheri* Sond., *C. moschata, C. maxima*, and *C. maxima* × *C. moschata* hybrids had no such effect on muskmelon cultivars Proteo and Supermarket (Trionfetti-Nisini et al., 2002). Finally, Park et al. (2013) examined four *C. melo* accessions and a Shintoza hybrid as rootstocks and found that none of these had an effect on the fruit weight of muskmelon (‘Earl’s elite’) and honeydew (‘Homerunstar’) except a *C. melo* accession (‘K134069’) which produced fruits of greater weight and size than the non-grafted control.

Other morphological traits of relevance to melon quality include fruit shape, exocarp and pulp thickness. Reports on melon grafting do not present significant rootstock effects on these variables which rather seem strongly delineated by the scion genotype. In the case of honeydew, muskmelon, cantaloupe and Piel de Sapo melons these traits were not affected by grafting on *Cucurbita* hybrid, *C. melo, C. maxima* and *C. melo* subsp. *agrestis* rootstocks (Traka-Mavrona et al., 2000; Fita et al., 2007; Colla et al., 2010a; Verzera et al., 2014). Similar results on fruit shape were obtained with Ananas and Galia type melons (Soteriou et al., 2016), and with cantaloupe grafted on hybrid rootstocks (Colla et al., 2006b), notwithstanding a limited increase in rind thickness.

##### Textural characteristics

Fruit texture is an essential characteristic for the organoleptic assessment of melon fruit and one often reported to deteriorate as a result of grafting (Rouphael et al., 2010). Grafting honeydew melon onto *Cucurbita* hybrids and *C. melo* rootstocks had no effect on fruit dry matter content and pulp firmness (Crinò et al., 2007). On the contrary, Colla et al. (2006b) found that pulp firmness values recorded for cantaloupe grafted on hybrid rootstock were significantly higher (19–32%) than those observed for non-grafted plants, and the same effect was observed with muskmelon grafted either on *C. melo* or *Cucurbita* hybrid rootstocks (Colla et al., 2010a), despite that grafting resulted in lower pulp dry matter content in both these studies. By contrast, flesh firmness of Galia melon was consistently reduced by grafting on four different hybrid rootstocks and a similar tendency was evidenced with Ananas melon; however, these results were obtained from graft combinations that demonstrated incompatibility problems, variably causing plant decline, with loss of pulp firmness being one of the quality indices proposed for prognostication of incompatibility (Soteriou et al., 2016). Galia melon grafted onto interspecific hybrid rootstocks and onto *C. metuliferus* incurred reduced overall sensory rating but not reduced flesh firmness compared to non-grafted controls (Guan et al., 2015), although the same scion grafted on *C. metuliferus* and grown organically in nematode infested soil incurred a reduction in pulp firmness (Guan et al., 2014).

Grafting interacted with scion cultivar in respect to firmness but a general trend for loss of firmness was identified in two annual field trials involving numerous cultivars of muskmelon, honeydew and specialty melon scions, although assessment of firmness across the various melon types was hampered by the difficulty of harvesting melons of the same maturity (Schultheis et al., 2015). Grafting cantaloupe on two interspecific hybrid rootstocks reduced flesh firmness compared to non-grafted and self-grafted control, with differences minimized after prolonged postharvest storage (Zhao et al., 2011). Finally, no effect on flesh firmness was reported when Piel de Sapo-type melon was grafted on either Monosporascus-resistant *C. melo* subsp. *agrestis* or the widely used but less compatible interspecific hybrid RS841 (Fita et al., 2007). From the findings above it is evident that, unlike the case of grafted watermelon (Kyriacou and Soteriou, 2015; Kyriacou et al., 2016), melon grafting, whether on same or different species rootstocks, unequivocally does not increase flesh firmness; it either has no effect or it results in loss of textural quality, depending largely on rootstock–scion compatibility and to a lesser extent on cultural conditions.

##### Sweetness and acidity

Fruit sweetness is a major sensory feature of melon quality (Yamaguchi et al., 1977; Liu et al., 2010), which stems mainly from soluble carbohydrates but is commonly quantitated on the basis of the SSC derived from the temperature-compensated refractive index of the fruit juice. Understanding rootstock-mediated effects on melon sweetness is critical for safeguarding sensorial acceptability of melon fruit produced on grafted plants. Available reports describing these effects manifest widespread rootstock–scion interaction attesting the importance of selecting appropriate graft combinations. For instance, grafting cantaloupe (cv. Cyrano) on hybrid ‘P360’ under greenhouse conditions reduced the fruit SSC by an absolute 1.6% when compared to the non-grafted control; however, the SSC (10.9%) remained highly acceptable (Colla et al., 2006b). Similar findings were reported for muskmelon (cv. Proteo) grafted on hybrid rootstock (‘P360’) in open field cultivation, though both grafted (8.3%) and non-grafted (7.5%) plants exhibited quite low fruit SSC (Colla et al., 2010b). However, evaluating the same scion (‘Proteo’) on hybrid rootstocks ‘P360’ and ‘PGM96-05,’ revealed no effect on SSC though grafting both ‘Proteo’ and ‘Supermarket’ muskmelons on *B. hispida* and *C. metuliferus* did reduce their SSC (Trionfetti-Nisini et al., 2002). Crinò et al. (2007) found no effect on the SSC of honeydew melon (cv. Incas) by grafting on four hybrids and four *C. melo* rootstocks, as also verified by Verzera et al. (2014) using the same scion on three hybrids and two *C. melo* rootstocks, but not with hybrid rootstock ‘AS10’ which increased the SSC from 15.5 to 16.3%. Galia melon ‘Arava’ grafted onto hybrids ‘Strong Tosa’ and ‘Carnivor’ incurred reduced overall acceptability, flavor rating and SSC compared to non-grafted controls, but not when grafted onto *C. metuliferus* (Guan et al., 2014, 2015). Moreover, honeydew scion (‘Honey Yellow’) on the same rootstocks did not differ in sensory properties and SSC in comparison with either non-grafted or self-grafted controls (Guan et al., 2014, 2015). The SSC of Piel de Sapo melon was slightly reduced when grafted on Monosporascus-resistant rootstock *C. melo* subsp. *agrestis* (‘Pat 81’) and also on hybrid ‘RS 841,’ but not to an extent that might significantly affect marketability (Fita et al., 2007). Although decrease in the SSC is not an infrequent response to grafting, with potential implications on, the increment of decrease in none of the reported studies seemed decisive for overall quality and marketability.

Besides the case of hybrid ‘AS10’ above, few are the cases of rootstocks reported of causing increase in melon SSC. One such is the absolute increase of 1.2% obtained in the mean SSC (10.4%) of greenhouse grown Galia melon grafted onto hybrid rootstocks ‘TZ148,’ ‘N101,’ ‘Carnivor,’ and ‘30900’ (Soteriou et al., 2016). Most often, grafting on compatible rootstocks has no effect on the fruit SSC. Out of four hybrid rootstocks onto which Ananas melon was grafted only one (‘30900’) had an effect on the SSC causing an absolute reduction by 1.12% relative to the non-grafted control (Soteriou et al., 2016). Field trials of muskmelon, honeydew and specialty melon types (Persian, Tuscan, Canary, Galia, Piel de Sapo) grafted on hybrid rootstock ‘Carnivor’ vs. self-grafted and non-grafted controls generally showed no effect on the SSC although limited grafting × scion interaction was evident (Schultheis et al., 2015). Traka-Mavrona et al. (2000) also found grafting had no effect on the fruit SSC of three inodorous melon and one cantaloupe cultivars tested on two hybrids and one pumpkin landrace rootstocks under protected and open field cultivation. Finally, in a study closely observing fruit harvest maturity based on the date of fruit setting, Park et al. (2013) reported that grafting both muskmelon (‘Earl’s elite’) and honeydew melon (‘Homerunstar’) onto four *C. melo* accessions and one Shintoza type *Cucurbita* hybrid had no effect on the scions’ fruit SSC, and they repudiated categorically claims of reduced fruit quality as a result of grafting, provided compatible rootstocks.

Further to the effect of grafting on SSC, the concentrations of soluble sugars are also critical as they dictate their relative contribution to the sweetness index (SI) of fruits (Elmstrom and Davis, 1981). According to Liu et al. (2010), accumulation patterns for hexoses, sucrose and oligosaccharides were similar during muskmelon ripening from non-grafted and grafted plants, despite differences in sugar levels between rootstocks. Moreover, during the period of fast sugar accumulation (32–48 days after anthesis) muskmelons from grafted plants maintained higher starch content than the non-grafted control, and the starch fraction was higher in the lower sugar content rootstock and lower in the non-grafted control. It was further postulated that the marked increase in mesocarp starch content may derive from competition by the vigorous rootstocks for the soluble sugars translocated to sink fruit which the rate of sucrose decomposition was unable to satisfy. Differential rootstock-mediated patterns of soluble sugars’ accumulation were depicted in a recent study by Soteriou et al. (2016), wherein total soluble sugars in the pulp of both Galia and Ananas melons were not differentiated between four hybrid rootstocks and the non-grafted control; however, grafting Galia generally increased fruit sucrose levels at the expense of fructose and glucose whereas the opposite was observed with Ananas melon (Soteriou et al., 2016).

Like most cucurbits, melon is a fruit of very low acidity, usually below 0.2% in citrate equivalents, which nevertheless affects the sweet-to-sour balance in sensory perception (Crinò et al., 2007; Colla et al., 2010b; Verzera et al., 2014; Guan et al., 2015). Grafting honeydew melon and cantaloupe on hybrid rootstocks had a minimal effect on fruit TA which was inconsequential to fruit sensory quality (Colla et al., 2006b; Verzera et al., 2014). Similarly, no effect was found on the TA of muskmelon and Galia melon by grafting on either *Cucurbita* hybrid or *C. melo* rootstock (Crinò et al., 2007; Colla et al., 2010b; Zhao et al., 2011; Guan et al., 2015).

##### Aroma profile

The production of volatile compounds in melon is associated with ethylene-dependent pathways (Obando-Ulloa et al., 2008; Pech et al., 2008) and with textural changes related to cell wall matrix solubilization events (Dos-Santos et al., 2013). Hence, the climacteric (*cantalupensis* and *reticulates*) and the non-climacteric (*inodorus*) types demonstrate distinct volatile profiles, with C9 aliphatic aldehydes being the key aroma and flavor descriptors for inodorous honeydew melons (Verzera et al., 2014), as opposed to the mainly ester-based (ethyl butanoate, methyl 2-methylbutanoate and ethyl 2-methylpropanoate) descriptors for fruity and sweet aroma notes of *cantalupensis* and *reticulates* muskmelon cultivars (Kourkoutas et al., 2006; Beaulieu and Lea, 2007). Grafting seems to affect the aroma profile of both muskmelon and honeydew type melons. Grafting muskmelon on interspecific *Cucurbita* hybrids and on *C. melo* rootstocks generally elicited higher levels of non-key alcohol and aldehyde volatile compounds responsible for green and fresh notes, such as flower-green (1-hexanol), fruity (2-methyl-1-butanol), fatty-green (1-octanol), ethereal (ethanol), green [(*E*)-2-butenal], and fresh-lemon-green (octanal) aromas (Condurso et al., 2012). Ester-based aromas characteristic of muskmelon were generally higher in non-grafted control, such as cantaloupe-like, green fruity, melon (ethyl 2-methylbutanoate) and sweet-fruit (ethyl butanoate) aromas (Chuan-qiang et al., 2011; Condurso et al., 2012). However, significant exceptions to this motif were found among both *Cucurbita* spp. and *C. melo* rootstocks, rendering screening for optimum rootstock–scion combinations essential. In fact, some commercial *Cucurbita* hybrids (e.g., ‘RS-841’) and *C. melo* (e.g., ‘Energia’) rootstocks can be successfully used for controlling soilborne pathogens without any significant effect on the fruit aroma (Condurso et al., 2012). Similarly, Verzera et al. (2014) examined the effect of four inter-specific hybrids and two melon genotypes on the fruit aroma and sensory quality of honeydew melon cv. Incas (*C. melo* L. subsp. *melo* var. *inodorus* H. Jacq.). Prevalent volatiles in both grafted and non-grafted *inodorus* melon were mainly aldehydes and alcohols such as nonanal (melon, orange peel), (*Z*)-6-nonenal and (E)-2-nonenal (honeydew melon fruity), (*E,Z*)-2,6-nonadienal, 1-nonanol (herbaceous), (*Z*)-3-nonen-1-ol (melon, green, floral) and (*Z,Z*)-3,6-nonadien-1-ol (pumpkin, cucumber). Fruits from plants grafted on three of the interspecific hybrids (cvs. RS-841, P-360, Polifemo) and one *C. melo* rootstock (‘Energia’) had similar aroma profiles to the control, however, particular rootstocks from either type (e.g., ‘AS10’ and ‘Sting’) were found to decrease the amounts of key aroma compounds. Grafting was generally found to reduce the intensity of honeydew melon and herbaceous aroma descriptors and increase those related to fruity aroma and flavor. It is important to emphasize that selection is possible of resistant interspecific hybrid rootstocks (e.g., ‘RS-841’) that increase yield and fruit weight of both honeydew cv. Incas and muskmelon cv. Proteo scions, without having a detrimental effect on sensory characteristics, including the aroma profile (Condurso et al., 2012; Verzera et al., 2014).

##### Functional compounds

Melon is a rich source of α-, ζ-, and especially β-carotene but also of lutein, cryptoxanthin, phytoene, and the violaxanthin cycle carotenoids, however, little is known on the effect of grafting on these components (Laur and Tian, 2011). The fruit carotenoid profile of odorous melon, was either non-differentiated, or highly improved particularly with regards to the α- and β-carotene components in response to grafting on *C. maxima × C. moschata* hybrid rootstocks vis-à-vis the non-grafted control; whereas grafting on *C. melo* rootstocks resulted in significantly reduced β-carotene levels, which inadvertently emphasized ζ-carotene content, while lutein was increased with grafting on both types of rootstocks (Condurso et al., 2012). Carotenoid content is largely responsible for melon pulp color; hence the effects of grafting on these traits are expectedly associated. Colla et al. (2006b) reported that grafting cantaloupe (cv. Cyrano) on hybrid rootstock ‘P360’ influenced pulp colourimetric values positively, resulting in brighter (higher L^∗^) and more intense orange hue (higher a^∗^/b^∗^ ratio), probably reflecting higher α- and β-carotene concentrations in the pulp (Condurso et al., 2012). Intriguingly, increased levels of both chlorophylls and β-carotene were obtained in the leaves of Galia type cvs. Arava and Resisto grafted on interspecific rootstocks ‘Shintoza,’ ‘Kamel,’ and particularly on ‘RS841’ (Romero et al., 1997).

#### Cucumber (*Cucumis sativus* L.)

Cucumber constitutes an annual vegetable species mostly grown under protected cultivation. The use of rootstocks resistant or tolerant to soilborne diseases, foliar pathogens, arthropods, and weeds has become instrumental for cucumber production, especially under intensive farming practices with limited crop rotations (Lee et al., 2010; Louws et al., 2010). Several rootstocks (*C. maxima* × *C. moschata*, *C. ficifolia*, *C. moschata*, *C. argyrosperma, L. siceraria*, *B. hispida*, *Luffa cylindrica* (L.) M. Roem., *Momordica charantia* L., *S. angulatus*, *Citrullus* spp.) have been used for cucurbit grafting; most enhance scion growth and productivity under unfavorable soil and environmental conditions, but some lack tolerance to specific stresses and others can have a detrimental effect on vegetable fruit quality (Rouphael et al., 2010, 2012). The most popular rootstocks for cucumbers belong to the genus *Cucurbita*. In particular, the interspecific cross *C. maxima* × *C. moschata* has been exploited as a favorable source of rootstocks, currently the most common commercial rootstocks for cucumber (Lee et al., 2010). Less frequent is the use of single non-hybrid *Cucurbita* species as rootstocks, such as accessions of *C. argyrosperma*, *C. ficifolia*, *C. maxima*, *C. moschata*, and *C. pepo*. Fruit quality deterioration in grafted plants, reported chiefly as decrease in sweetness and acidity, is a common problem particularly with *Cucurbita* hybrids which are frequently implicated in scion × rootstock interactions, further compounded by crop management practices (Davis et al., 2008b; Rouphael et al., 2010).

##### Morphometric characteristics

It is well established that vigorous *Cucurbita* interspecific hybrids can improve cucumber yields significantly (Davis et al., 2008b). More frequently, the effect on yield is related to the variation in fruit size, as grafted plants are characterized by a vigorous root system (high root length and density) able to enhance photosynthetic rate as well as water and nutrient uptake efficiency (particularly N, P, Ca, and Mg) and, consequently, crop productivity (Rouphael et al., 2010). Several authors have demonstrated a significant increase in fruit weight when cucumber plants were grafted onto *Cucurbita* interspecific hybrids (‘RS841,’ ‘Strong Tosa,’ ‘PS1313,’ and ‘P360’) and *Cucumis pustulatus* Naudin ex Hook.f. compared to non-grafted control (Colla et al., 2012, 2013; Goreta Ban et al., 2014; Liu et al., 2015). However, in some cases increased cucumber yield has been attained mainly by an increase in the number of fruits per plant rather than an increase in mean fruit size (Huang et al., 2009).

Other morphological traits that constitute primary criteria for making purchasing decisions are the fruit shape index and the colouration of the skin (Rouphael et al., 2010). Reports on cucumber grafting demonstrated that the effect of rootstocks on fruit shape has been mostly non-significant or minimal (Lee et al., 1999; Colla et al., 2013). Regarding color, Colla et al. (2012) reported that lightest colouration, expressed as an increase in colourimetric CIELAB component L^∗^, was observed on the skin of cucumber cv. Akito grafted onto the commercial rootstock ‘PS1313’ (*C. maxima* × *C. moschata*) compared to fruit from plants grown on their own roots.

##### Textural characteristics

Fruit firmness constitutes also an important physical property influencing consumer acceptability (Rouphael et al., 2010). Hwang et al. (1992) demonstrated that cucumber from plants grafted onto *S. angulatus* ‘Andong’ rootstock tended to be firmer than those grafted onto figleaf gourd (*C. ficifolia* ‘Heukjong’). On the contrary, Morishita (2001) reported that ‘Kema’ and ‘Kifujin New Type’ cucumber plants grafted onto the bloomless rootstock ‘Big Ben Kitora’ carried fruits of softer flesh than those grown on their own roots. Nevertheless, more popular rootstocks such as *C. moschata* or *C. maxima* × *C. moschata* had no effect on fruit firmness when compared to non-grafted plants (Sakata et al., 2007; Colla et al., 2013). The variation in fruit firmness induced by rootstocks may be attributed to several mechanisms such as the uptake and translocation of calcium, modulated water relations and nutritional status, increased synthesis of endogenous hormones as well as variation in cell morphology and turgor (Rouphael et al., 2010).

##### Sweetness and acidity

It has been reported that changes in grafted vegetable aroma and taste appear to be not only scion but also rootstock-dependent attesting the importance of selecting appropriate graft combinations (Rouphael et al., 2010). For instance, grafting cucumber onto ‘Heukjong’ figleaf gourd (*C. ficifolia*) reduced the fruit SSC and fructose concentration when compared to the non-grafted control; whereas the SSC remained high when ‘Andong’ (*S. angulatus*) was used as rootstock (Lee et al., 1999). Moreover, Huang et al. (2009) observed a lower accumulation of SSC in plants grafted onto figleaf gourd and grown under unstressed conditions compared to self-grafted control. However, under saline conditions of 60 mM NaCl the SSC and TA incurred significant increase in both grafted and self-grafted plants. Similarly, the SSC of ‘Akito’ cucumber increased when grafted onto the interspecific hybrid ‘PS1313,’ whereas an opposite trend was recorded for the TA (Colla et al., 2013).

##### Aroma profile

Besides taste, the effect of grafting on aroma was also quantitated in cucumber. In a recent study, Guler et al. (2013) demonstrated that grafting affected the aroma profile of both the peel and flesh of cucumber cv. Cengelköy in response to the use of a bottle gourd rootstock. Thus, grafting caused a substantial increase in the alcohol content [(*Z*)-6-Nonenol, (*E,Z*)-2,6-nonadienol, 1-nonanol, and (*Z,Z*)-3,6-nonadienol], a decrease in the aldehyde content [(*E,Z*)-2,6-nonadienal] with no significant influence on ketones, terpenes and hydrocarbons in both cucumber peel and flesh tissues. The authors concluded that the bottle gourd ‘33-41’ could be considered a promising rootstock for improving major volatile components identified in cucumber.

### Solanaceae

#### Tomato (*Solanum lycopersicum* L.)

Nowadays tomato production under protected environment is resorting to the use of grafted plants. Reservations against their use relate to their higher price being considered non-affordable by growers, the use of speciality cultivars or particular problems associated with disease outbreaks, such as the novel tobamoviruses (Luria et al., 2017). However, the number of commercial rootstocks offered by breeding companies has burst and their widespread use is becoming highly visible. Not only rootstocks from the species *Solanum lycopersicum* L. are available but also interspecific hybrids, such as *S. lycopersicum* × *Solanum habrochaites* S. Knapp & D.M. Spooner, and rootstocks from other species, such as *Solanum torvum* L. or *Solanum melongena* L. Therefore, growers often follow breeders’ recommendation and use same-company scions and rootstocks. Even in the field, the application of grafted plants has started, e.g., as a means to protect the scion from invasive weeds, such as broomrape. Compared to the Cucurbitaceae representatives, the effect of rootstocks on fruit quality traits seems less intense and reports on reduced quality are mainly related to reduced sweetness. The effects of grafting on most tomato quality characteristics have been variable, strongly influenced by the rootstock–scion combination. Moreover, the effect of the grafting combination interacts with other factors, such as climate, cultural practice, duration and intensity of stress, water and nutrient disposability and not to least with the sampling strategy (Riga, 2015; see chapter ‘Methodological approaches’).

##### Morphometric characteristics

Grafting tomato often results in significant increase in fruit weight and consequently in fruit diameter and size compared with non- or self-grafted plants (Passam et al., 2005; Moncada et al., 2013; Riga, 2015). This was reported for many different rootstock–scion combinations resulting in total yield increase. However, yield gain may be also attributed to an increase in the number of fruits rather than an increase in mean fruit weight (Savvas et al., 2011). The effect of grafting on fruit weight and size depends on grafting combinations (Khah et al., 2006; Leonardi and Giuffrida, 2006; Schwarz et al., 2013). Larger fruit size seems to be attained when vigorous rootstocks are used, such as ‘Maxifort’ (Krumbein and Schwarz, 2013; Schwarz et al., 2013), ‘Beaufort’ (Romano et al., 2000; Pogonyi et al., 2005; Turhan et al., 2011), ‘Heman’ (*S. habrochaites*), ‘Joint,’ ‘P1614,’ and ‘RS1427’ (Romano et al., 2000), or ‘Star Fighter’ (Theodoropoulou et al., 2007). This phenomenon is particularly recognized when scions have smaller fruit sizes, e.g., cherry tomato with less than 40 g (Schwarz et al., 2013). In some cases, grafting may reduce fruit size when less vigorous rootstocks are used, such as ‘Brigeor’ (Schwarz et al., 2013), ‘Energy,’ ‘Firefly,’ ‘Linea9243,’ ‘Nico’ (Romano et al., 2000). Based on the same reasons, fruit size of two different scion cultivars was significantly reduced when a salt tolerant goji berry (*Lycium chinense* Mill.) served as rootstock (Huang et al., 2015).

Fruit shape has seldom been assessed in grafted tomato despite indications of its differentiation once the fruit size is affected (Schwarz et al., 2013). Increase in shape index, measured as the ratio of fruit diameter to maximal height, was reported as corresponding to increase in fruit size (Turhan et al., 2011). However, rootstock ‘Beaufort’ raised the fruit shape index, compared to non-grafted tomato, irrespective of the scion cultivar (‘Yeni Talya,’ ‘Swanson,’ ‘Beril’), while rootstock ‘Arnold’ only increased it in combination with the scion ‘Yeni Talya.’ This indicates a similar dependence of fruit shape to rootstock vigor as already mentioned for the fruit size.

Fruit color was in certain cases affected by grafting (Di Gioia et al., 2010; Brajović et al., 2012) but in others not (Krumbein and Schwarz, 2013; Schwarz et al., 2013). As in the case of watermelon (see chapter ‘Watermelon’), changes pertained particularly to color component a^∗^ (redness) which is associated with lycopene content. Thus, also for tomato, an effect of grafting on color seems to be significant if a rootstock influences the fruit lycopene content (Miskovic et al., 2016). However, color as well as texture assessment, are often presented with the difficulty of obtaining sufficient and uniform fruit samples in terms of development and harvest maturity to constitute a representative sample. Failure to control sampling procedures effectively may lead to misleading or inconsistent results (see also chapter ‘Methodological approaches’).

##### Physiological defects

Although physiological disorders related to grafted plants have not received much attention in the literature, they are not uncommon. Blossom end-rot (BER), the most typical tomato disorder (Ho and White, 2005), was invariably reduced in tomato grafted on rootstocks ‘Brigeor,’ ‘Maxifort,’ and LA1777 (*S. habrochaites*), and under different environmental conditions shaped by factors such as salinity, potassium nutrition, sub-optimal temperature, and light conditions (Fan et al., 2011; Krumbein and Schwarz, 2013; Schwarz et al., 2013; Ntatsi et al., 2014). Moreover, reduction in BER incidence with grafting was pronounced under stress conditions in comparison to both non- and self-grafted scions. Decrease in BER was mainly related to the rootstock genotype; e.g., the BER incidence in ‘Classy,’ a medium round type tomato of ∼70 g, was more diminished when grafted on the rootstock ‘Brigeor’ compared to ‘Maxifort’ or self-grafted plants. The BER reduction was also influenced by rootstock–scion interaction; e.g., it was decreased to a greater extent when cherry tomato ‘Piccolino’ was used as a scion. Under certain conditions, the use of a rootstock may raise the BER incidence. This was the case in an experiment where two cultivation systems were compared during summer: hydroponics vs. soil (Takasu et al., 1996); the improved nutrient and water uptake facilitated by grafting did not cope sufficiently with the very fast fruit growth under high radiation conditions. Also, BER increased in trials involving rootstock ‘Edkawi,’ or eggplant rootstocks (e.g., ‘EG203,’ ‘VFR Takii’) (Oda et al., 1996; Poudel and Lee, 2009; Fan et al., 2011). Here possible reasons are justified by the characteristics of the rootstocks selected. Rootstock ‘Edkawi,’ although known as salinity-tolerant, as well as eggplant rootstocks, lower the uptake and transport of Ca-ions into the fruits compared with self-grafted tomato. Results indicate that the incidence of BER is reduced by grafting when Ca uptake and transport into the fruits is improved (Fan et al., 2011; Savvas et al., 2017). Increased fruit Ca concentration may lead to strengthening of cell walls and cellular integrity and improvement of fruit firmness (Dorais, 2007; Schwarz et al., 2013).

##### Textural characteristics

Attributes of texture are seldom considered in grafted tomato. Cultivar Jack grown under Mediterranean conditions as a scion grafted onto nine rootstocks typified rootstock effects: e.g., ‘Alligator’ tended to reduce, ‘Maxifort’ did not affect and ‘King Kong’ enhanced firmness (Riga, 2015). Other reports corroborate these findings although loss of firmness seems as the predominant effect. Thus, fruits of the cultivars ‘Classy’ and ‘ASVEG10’ obtained from plants grafted onto ‘Brigeor’ or ‘Maxifort’ and grown under potassium deficiency but also fruits from plants grafted on eggplant rootstock were less firm and scored higher maximum deformation than fruits from self-grafted tomato (Poudel and Lee, 2009; Schwarz et al., 2013). The reasons are not clear but K^+^/Ca^2+^ interaction was not implicated in the differences in fruit firmness. Independently, it could be clearly demonstrated that fruit Ca content was increased by grafting (Khah et al., 2006; Fan et al., 2011; Savvas et al., 2017). However, as in the case of Khah et al. (2006) it did not affect fruit firmness. While Riga (2015) did not find differences in fruit firmness between non- and self-grafted ‘Jack’ tomato, Rahmatian et al. (2014) found significantly lower firmness in fruits from self-grafted compared to non-grafted ‘Synda’ tomato. However, the use of a rootstock (cv. King Kong) independently of simple or double grafting did not affect firmness.

##### Sweetness and acidity

Results concerning the variation in taste of grafted tomato fruit, comprising sugars (glucose, fructose), SSC as a non-specific sweetness parameter, and TA are also very contradictory and seem to be affected by the same parameters as mentioned above. In several experiments, the use of a rootstock did not change fruit taste attributes (Matsuzoe et al., 1996; Khah et al., 2006; Savvas et al., 2011; Barrett et al., 2012). However, decrease and increase in the main components of taste were also observed, as explained below, and based on these findings grafting appears not to constitute a reliable tool for improving tomato fruit taste. This conclusion was confirmed by the results of one of the rare consumer sensory tests performed during a 2-year cultivation of the heirloom tomato ‘Brandywine’ as non-, self-grafted and grafted onto ‘Survivor’ and ‘Multifort.’ While in the first year the rootstock ‘Survivor’ scored significantly lower than the non-grafted ‘Brandywine’ in appearance, acceptability, and flavor, no differences were observed between these treatments in the second year (Barrett et al., 2012).

The main sugars in mature tomato are glucose and fructose in equal shares and the total sugar concentration ranges from about 20 to 100 g⋅kg^-1^ fresh mass, depending on cultivar and growing conditions. Improvement of fruit sweetness related to grafting is rather seldomly reported. Such cases described were with tomato grafted onto ‘Fanny,’ ‘King Kong,’ ‘LA1777’ (*S. habrochaites*), or onto scarlet eggplant rootstocks (e.g., ‘EG 203’), whereby the enhanced SSC content was associated with the effect of water deficiency which lowered plant growth and yield and decreased fruit water content (Oda et al., 1996; Fernández-García et al., 2004a,b; Poudel and Lee, 2009; Ntatsi et al., 2014; Rahmatian et al., 2014). The same association occurred when grafted plants grew under saline or drought conditions or when using a drought tolerant cultivar as a rootstock (Flores et al., 2010; Sánchez-Rodríguez et al., 2012a) or when grafting onto a medicinal plant (*L. chinense*; Huang et al., 2015). However, in many grafting combinations, rootstocks reportedly decreased SSC and sugar concentration in the scion fruits (Pogonyi et al., 2005; Qaryouti et al., 2007; Turhan et al., 2011; Barrett et al., 2012; Nicoletto et al., 2013a,b; Schwarz et al., 2013; Gajc-Wolska et al., 2015; Kumar et al., 2015; Riga, 2015). Nevertheless, the decline caused by grafting is very low compared to the potential increase procured by employing a selected scion that might at least double the fruit sugar concentration. The decline in sugars incurred with grafting is reported to account for approximately not more than 16% (Riga, 2015), which does not exceed the range of maximum decline proposed for consumer acceptability (Kader, 1999; Maynard et al., 2002). The reasons for a lower carbohydrate content in grafted tomato may stem indirectly through rootstock effect on scion vigor, timing of flowering, fruit load, yield and, ultimately, fruit maturation, as fruit sugar concentration is highly dependent on fruit maturity at harvest (Rouphael et al., 2010; Soteriou and Kyriacou, 2015). In this respect, grafting may be considered a high-input production method, with a prevalent tendency for increasing crop load and potentially suppressing fruit sugar content (Davis et al., 2008b; Soteriou and Kyriacou, 2015). Moreover, vigorous rootstocks may act as additional sinks for assimilates and thus, reduce assimilate flow to the fruits (Xu et al., 2006; Martínez-Ballesta et al., 2010). Alternatively, water uptake-efficient rootstocks may increase fruit water content even if sufficient assimilates are available, thus, leading to a reduced fruit sugar concentration (Turhan et al., 2011; Krumbein and Schwarz, 2013). Fruits of grafted scions are often larger than fruits of the same non- or self-grafted scion, and though the fruit sugar/acid ratio might remain unaffected, a decline in soluble carbohydrates may be incurred as a dilution effect (Tieman et al., 2017).

While sugars may decrease in grafted tomato, acid content expressed as TA is on the contrary enhanced (Turhan et al., 2011; Sánchez-Rodríguez et al., 2012a; Nicoletto et al., 2013a,b; Schwarz et al., 2013; Krumbein and Schwarz, 2013; Huang et al., 2015; Kumar et al., 2015; Riga et al., 2016). Total organic acids in tomato fruit are usually in the range of 0.2 to 1.7 g⋅kg^-1^ fresh mass, with citric and malic acid being the main components of sourness. Grafting accounts for an increase in TA up to 15% reported under a range of different environmental conditions which indicates a direct rootstock effect. Comparing fruits from ‘Classy’ and ‘Piccolino’ self-grafted or grafted onto ‘Maxifort’ and ‘Brigeor’ resulted under different experimental conditions almost always in the highest TA produced by ‘Maxifort’ followed by ‘Brigeor’ and self-grafted. Independent of the presence or absence of water stress, tomato fruits from the drought sensitive cultivar Josefina grafted onto the drought tolerant cv. Zarina had always higher TA contents, particularly citric acid, compared with non-, self-grafted or the reciprocal grafting combination (Sánchez-Rodríguez et al., 2012a).

The mechanisms involved in grafting-elicited increase of fruit TA have not been thoroughly investigated; however, organic acids constitute a direct substrate for respiratory demands and their increased *de novo* synthesis in developing fruits might be a plausible mechanism for coping with the sugar deficit incurred on the heavy crop load supported by vigorous rootstocks. Moreover, the capability of a vigorous root system to enhance the uptake of nutrients, such as K, could be another reason (Ruiz and Romero, 1999; Leonardi and Giuffrida, 2006; Albacete et al., 2009). Potassium is positively related to the acid concentration in tomato fruits, and plays a role in maintaining electroneutrality of acids in the fruit. However, K transport depends not only on the rootstock but also on growing conditions, such as the current K concentration in the root zone, and on climatic factors (Albacete et al., 2009). Interestingly, differences in K concentration were found not to be significant between fruits of self-grafted and grafted plants, but increase in fruit TA was significant (Schwarz et al., 2013). To complicate matters further, an exceptional report demonstrated that when cv. Lemance was grafted onto ‘Beaufort,’ fruit organic acid concentration was lower compared with fruits from non-grafted plants (Pogonyi et al., 2005). Therefore, the enhanced TA in fruits of grafted tomato warrants further investigation (Leonardi and Giuffrida, 2006).

##### Aroma profile

Odor-active volatiles contribute to tomato flavor (Krumbein and Auerswald, 1998) but their influence on sensory properties awaits further treatise. Six major volatiles which contribute to tomato flavor were evaluated by a consumer panel in Florida (USA): 2-butylacetate, *cis*-3-hexen-1-ol, 3-methyl-1-butenol, 2-methylbutanal, 1-octen-3-one, *trans*,*trans*-2,4-decadienal (Tieman et al., 2012; Zhang B. et al., 2016). Krumbein and Schwarz (2013) found that for two different scion cultivars, ‘Piccolino’ – a cherry, and ‘Classy’ – a round type, grafting on rootstocks ‘Brigeor’ and ‘Maxifort’ induced a general enhancement of three aroma volatiles: methyl salicylate, guaiacol and eugenol, with oily, sweet and spicy odors, respectively; but the concentrations of three other aroma volatiles with almondy odor (benzaldehyde), violet-like odor (β-ionone) and tomato-like flavor (geranylacetone) were decreased by grafting. The variation of the carotenoid content in tomato (see below) affects the carotenoid-derived volatiles responsible for tomato flavor, such as the violet-like odor (β-ionone) and the tomato-like flavor (geranylacetone) (Krumbein and Schwarz, 2013). However, the actual sensory contribution of these volatiles to changes in tomato flavor was not assessed, which remains critical particularly under the light of the findings of Tieman et al. (2012).

##### Functional compounds

Carotenoid content of tomato fruit, mainly lycopene and β-carotene, can be influenced by grafting, but it is subject to significant rootstock–scion interaction which indicates that graft combination plays an important role. Moreover, as Riga et al. (2016) demonstrated, the comparison of the grafting combination to either the non-grafted or self-grafted scion is very important. According to several authors, lycopene concentration in tomato fruits tends to decrease with grafting (Helyes et al., 2009; Brajović et al., 2012; Nicoletto et al., 2013b); e.g., most out of 15 rootstocks investigated, including ‘Maxifort,’ ‘Beaufort,’ and ‘King Kong,’ decreased the fruit lycopene concentration of tomato scion ‘Jeremy’ and ‘Jack’ (Miskovic et al., 2009; Riga et al., 2016). Similar results have been reported for tomato scion ‘Cecilia’ grafted onto ‘Beaufort’ and ‘Heman’ (Mohammed et al., 2009), for scion ‘Macarena’ grafted onto ‘Maxifort’ (Gajc-Wolska et al., 2010), as well as for ‘Classy’ grafted onto ‘Brigeor’ (Krumbein and Schwarz, 2013; Schwarz et al., 2013). Though in the latter experiments total carotenoids were diminished due to lycopene decrease, under specific conditions of grafting the lycopene/carotenoid content may increase. When eggplant rootstock ‘Madonna’ was used, tomato lycopene concentration increased (Miskovic et al., 2016). In another experiment, nutritional stress caused by low potassium in the nutrient solution was applied to ‘Classy’ grafted onto ‘Maxifort’ resulting in enhanced β-carotene (Schwarz et al., 2013), as well as in another grafting combination (‘Amati’ grafted onto ‘Robusta’ or ‘Body’) under non-stressed conditions (Brajović et al., 2012). The mechanisms for these opposite responses remain unclear. In other studies, authors did not find any grafting effect on carotenoids (Khah et al., 2006; Vinkovic-Vrcek et al., 2011) of ‘Big Red’ tomato grafted onto ‘Heman’ and ‘Primavera’ (*S. lycopersicum*) under open-field and greenhouse conditions and of ‘Tamaris’ grafted onto ‘Heman,’ ‘Efiato,’ and ‘Maxifort’ always comparing with fruits from non-grafted cultivars.

Tomato fruit contains significant amounts of ascorbic acid, and several studies showed that fruit content strongly reduced by grafting both in greenhouse and field studies (Fernández-García et al., 2004a,b; Arvanitoyannis et al., 2005; Di Gioia et al., 2010; Vinkovic-Vrcek et al., 2011; Djidonou et al., 2016; Riga et al., 2016). Fruit vitamin C content was reduced in soil cultivation of different tomato scions grafted onto ‘Heman,’ ‘Spirit,’ ‘Arnold,’ ‘Beaufort’ (Qaryouti et al., 2007; Turhan et al., 2011) and in hydroponics using ‘Maxifort,’ ‘Interpro,’ or ‘King Kong’ rootstocks (Riga et al., 2016). The lower ascorbic acid content could be explained by the higher plant/shoot biomass in grafted plants compared with non-grafted ones or by the fact that grafted plants were initially subjected to stress following the grafting operation. Ascorbic acid is known to control cell differentiation (Arrigoni, 1994) and to promote callus division and growth (Tabata et al., 2001). The decreased total vitamin C content of the fruits from grafted plants could therefore be a resultant of redistribution or accumulation of vitamin C in other parts of grafted plants (Wadano et al., 1999). Alternatively, changes in ascorbic acid content can be influenced by the choice of rootstock, as shown for tomato grafted onto ‘King Kong,’ ‘Beaufort,’ or ‘Maxifort’ rootstocks, which exhibited higher ascorbic acid content compared to the same plants self-grafted or grafted onto ‘Arnold’ and ‘Brigeor’ rootstock, respectively (Turhan et al., 2011; Schwarz et al., 2013; Rahmatian et al., 2014). A similar increase in Vitamin C was analyzed when tomato were grafted on *L. chinense* (Huang et al., 2015).

Abundant flavonoids in tomato fruits are the hydroxycinnamic acids and their derivatives (Gómez-Romero et al., 2010; Sánchez-Rodríguez et al., 2012b; Riga et al., 2016), as well as naringenin, chalcone and rutin (quercetin-3-*O*-rutinoside) (Slimestad et al., 2008; Sánchez-Rodríguez et al., 2012b), which are natural antioxidants. The choice of cultivar (Steward et al., 2000) as well as abiotic and agronomic factors are major contributing factors to the total content of phenolics in tomato (Tomas-Barberan and Espin, 2001). Under water stress the combination with a drought tolerant rootstock (cv. Zarina) resulted in the highest value in total flavonoids, hydroxycinnamic acids and rutin compared with not or self-grafted ‘Zarina.’ Nicoletto et al. (2013b) found also a higher phenolic acid content for another grafting combination with ‘Profitto’ grafted onto ‘Beaufort’ compared with non-grafted plants, but this was not observed with rootstock ‘Big Power.’ However, Vinkovic-Vrcek et al. (2011) reported that grafting significantly reduced the total phenolic content of tomato cv. Tamaris grafted onto ‘Heman,’ ‘Efiato,’ and ‘Maxifort,’ while no significant differences were found among these rootstocks. Comparing nine different mainly commercial rootstocks, Riga et al. (2016) confirmed that the reduction or increase in flavonoids clearly depends on the selection of the rootstock when the same scion cultivar was used. Thus, relative to tomato from non-grafted ‘Jack,’ soluble and total phenolics were reduced when grafted onto ‘King Kong’ but increased when grafted onto ‘Brigeor.’ The trigger for the rootstock to affect flavonoid concentration remains unclear. Although as indicated by the drought experiment, rootstocks better adapted to stress conditions responsible for higher flavonoid production may improve total flavonoids in the whole plant (Sánchez-Rodríguez et al., 2012b).

Among other functional compounds, serotonin concentration in fruits was found lower after grafting ‘Jack’ onto different commercial rootstocks independent of the cultivars selected (Riga et al., 2016).

#### Eggplant (*Solanum melongena* L.)

Eggplant and its relatives constitute an important source of rootstocks for the production of not only eggplant itself but also of tomato. By far the most common rootstock for eggplant is *S. torvum* (Lee et al., 2010). However, numerous other rootstock species and interspecific hybrids have also been tested as rootstocks for eggplant, including *S. incanum*, *S. incanum* × *S. melongena*, *S. melongena* × *S. aethiopicum*, *S. macrocarpon*, *S. sisymbriifolium*, *S. torvum* × *S. sanitwongsei*, *S. integrifolium* syn., *S. aethiopicum* gr. *Aculeatum* × *S. melongena*, S. *lycopersicum*, *S. lycopersicum* × *S. lycopersicum*, *S. habrochaites*, *S. lycopersicum* × *S. habrochaites* and *S. melongena* (Lee et al., 2010; Gisbert et al., 2011a,b; Khah, 2011; Moncada et al., 2013; Marsic et al., 2014; Sabatino et al., 2016). Current reports on the changes conferred by grafting on eggplant fruit quality provide conflicting information. This could be attributed in part to the environment in which experiments were ran (greenhouse vs. open-field), possible rootstock–scion interaction underscoring graft combinations, and differences stemming from failure to standardize fruit harvest maturity (Rouphael et al., 2010; Kyriacou et al., 2016).

##### Morphometric characteristics

Based on recent studies, the effect of grafting on eggplant mean fruit weight tends to be non-significant, compared to non- and self-grafted plants. For instance, when cultivar Black Beauty was cultivated non-grafted, self-grafted or grafted onto *S. torvum*, *S. incanum* × *S. melongena* and *S. melongena* × *S. aethiopicum* similar mean fruit weights were observed (Gisbert et al., 2011b). Similar findings were also recorded when *S. melongena* landraces ‘Bianca,’ ‘Sciacca,’ ‘Marsala,’ and ‘Sicilia’ were grafted onto *S. torvum* under open field conditions (Sabatino et al., 2016). Khah (2011) also confirmed these results when eggplant cv. Rima was cultivated non-grafted, self-grafted, or grafted onto two hybrid tomato rootstocks, ‘Heman’ and ‘Primavera’ under both greenhouse and open-field conditions. Exceptional increase (29%) in fruit weight was reported for eggplant ‘Black Bell’ when grafted onto *S. torvum* and grown in a soilless system (Cassaniti et al., 2011).

Eggplant fruit shape is highly heritable and subject to strong genetic control (Gisbert et al., 2011b). Several studies revealed that the effect of grafting on shape index has been circumstantial and mostly non-significant or minimal (4%) when the following rootstocks were used: *S. incanum*, *S. incanum* × *S. melongena* and *S. torvum* (Cassaniti et al., 2011; Gisbert et al., 2011a,b). Information on fruit physical properties of grafted eggplants, such as peel color, is conflicting but generally considered as having a negative effect (Moncada et al., 2013). For instance, the calyx of ‘Brigah’ fruits from non-grafted plants exhibited higher values of lightness (L^∗^) and more vivid color saturation (chroma) in comparison to those from plants grafted onto *S. torvum*; however, in other similar works such differences between fruits of grafted and non-grafted plants were not observed (Cassaniti et al., 2011; Gisbert et al., 2011b). The most likely source of this disparity could be the difficulty of standardizing sampling practices based on optimal harvest maturity for eggplant.

##### Textural characteristics

Negative effects on eggplant fruit textural properties amounting to loss of firmness were reported when the *S. melongena* cultivars Black Bell and Tsakoniki were grafted onto *S. torvum* and *S. sisymbriifolium* rootstocks, respectively (Arvanitoyannis et al., 2005; Cassaniti et al., 2011). The greater fruit external and pulp internal firmness of non-grafted plants observed by Arvanitoyannis et al. (2005) could be attributed to the fact that the pest and disease pressures were more pronounced in this treatment. Therefore, it is likely that restriction of water uptake efficiency in non-grafted plants resulted fruits with lower water content and tougher texture.

##### Sweetness and acidity

Information on taste compounds of eggplant fruits in relation to grafting remains conflicting and conclusive trends may be difficult to deduce currently, however, the reporting of positive effects is the one mostly absent. For example, according to Lee et al. (2010) *S. torvum* rootstock had no effect on eggplant fruit sugar content. Moreover, only non-significant differences in the SSC, in TA, and in juice pH were recorded among fruits from non-grafted, self-grafted and plants grafted onto *S. habrochaites* and *S. lycopersicum* rootstocks (Khah, 2011). In line with the previous work, Arvanitoyannis et al. (2005) observed that grafted plants yielded less sweet fruits with lower ratings of sensory acceptability than non-grafted plants. The reduced fruit sugar concentration in the fruits of grafted plants may be attributed to several mechanisms, including (i) the reduction of assimilate flow to the reproductive organs since vigorous rootstocks may act as additional sinks for assimilates, and (ii) the increased water uptake by rootstocks which could reduce fruit dry matter content and consequently sugar content (Martínez-Ballesta et al., 2010; Rouphael et al., 2010).

##### Functional compounds

Eggplant is among the most important vegetables in terms of oxygen radicals scavenging capacity, which is a quality trait associated with its high content of phenolic antioxidants (Cao et al., 1996). Gisbert et al. (2011b) observed a higher total phenolic content only in fruits of eggplant ‘Cristal’ grafted onto *S. macrocarpon* rootstock. Furthermore, Sabatino et al. (2016) showed that grafting eggplant onto *S. torvum* increased total polyphenol fruit content in three out of four Sicilian landraces grown under open-field conditions, whereas an opposite trend was observed by Moncada et al. (2013), wherein the total phenolic content was greater in the non-grafted plants. Moreover, changes in fruit phenolic contents and other important flavonoids, notably anthocyanins, can be highly influenced by the rootstock–scion combination which is often subject to significant interaction (Marsic et al., 2014). However, the latter study also highlighted the importance of environmental parameters such as solar radiation in the same respect, as fruits from the same landrace/rootstock combination behaved differently in two growing seasons, with the first season being characterized by lower solar radiation compared to second. The higher vigor of grafted plants may have a negative effect on the concentration of anthocyanins, therefore grafted plants should be properly pruned under low solar conditions to improve light interception since the accumulation of anthocyanins in eggplant fruit epidermis is strongly dependent on light exposure (Awad et al., 2001).

#### Pepper (*Capsicum annuum* L.)

Pepper is currently the least grafted among the solanaceous crops, especially compared to tomato and eggplant, presumably because the commercial rootstocks currently available provide modest benefits (Lee et al., 2010). Accordingly, an urgent need exists for developing new rootstocks that can augment efforts to meet growing demands for fresh sweet pepper. The most popular rootstocks currently used are intraspecific hybrids or cultivars of *C. annuum*, however, accessions of the cultivated *Capsicum* species, including *C. baccatum* L., *C. chacoense* Hunz., C. *chinense* Jacq., and *C. frutescens* L. and their interspecific hybrids *C. annuum* × *C. chinense*, have also been tested as rootstocks for pepper (Lee et al., 2010). As might be expected, the main reason for grafting pepper has been the resistance to soilborne pathogens and nematodes but also to abiotic stresses (Schwarz et al., 2010; Penella et al., 2016), and very limited work has yet been conducted to address the implications of grafting for pepper fruit quality.

##### Morphometric characteristics

Several reports in the scientific literature indicated strong rootstock specificity in the responses of pepper to grafting (Rouphael et al., 2010). For instance, Doñas-Uclés et al. (2014) demonstrated an increase in fruit weight when cv. Palermo was grafted onto the *C. annuum* rootstock ‘Tesor,’ whereas the use of rootstocks ‘Oscos’ and ‘AR40’ incurred a minimal increase in fruit weight. Similarly, Leal-Fernández et al. (2013) showed that the mean fruit weight was higher when sweet pepper ‘Triple star’ was grafted onto chili pepper rootstock ‘AR96029,’ in comparison to non-grafted plants. In the case of F1 hybrids ‘Edo’ and ‘Lux,’ fruit weight was not influenced by grafting onto *C. annuum* rootstocks of the cultivars Snooker, Tresor, RX360, DRO8801, and 97.9001 (Colla et al., 2008). By contrast Gisbert et al. (2010) identified a general trend for reduction of fruit weight in greenhouse trials of grafted pepper (cvs. Almuden and Coyote), based, however, only on two hybrid rootstocks, ‘Charlot’ and ‘Foc.’ Decrease in fruit weight against non-grafted control is usually an indicator of rootstock–scion incompatibility.

Moreover, absence of defects in particular blossom end rot (BER) is another important quality consideration for peppers. This physiological disorder of the pepper fruit could be ascribed to a local shortage of Ca and is manifested as a leathery brown patch at the blossom-end of the fruit. However, to date there is no information in the international literature on whether the incidence of BER is reduced by grafting. The incidence of BER in grafted pepper plants could be influenced positively by rootstocks able to improve uptake and translocation of Ca to the fruits, thus strengthening cell walls and cellular integrity, or could be exacerbated by vigorous rootstocks of high nitrogen-uptake efficiency that may encourage fast growth whose demands in calcium might be difficult to meet. Therefore, research in this field is currently a prime necessity.

##### Sweetness and acidity

The SSC and TA of pepper fruit is in general not highly compromised by grafting on most commercial *C. annuum* rootstocks (Colla et al., 2008; López-Marín et al., 2013). In the former, two studies neither SSC nor TA were affected when pepper plants, cultivated under greenhouse conditions, were grafted onto the following *Capsicum* rootstocks: ‘Snooker,’ ‘Tresor,’ ‘RX360,’ ‘DRO8801,’ ‘97.90001,’ ‘Atlante,’ ‘Creonte,’ and ‘Terrano’ (Colla et al., 2008; López-Marín et al., 2013). Contrarily, positive effects were observed in the TA and SSC of ‘Herminio’ grafted onto ‘Atalante’ under both full and deficit irrigation conditions (López-Marín et al., 2017). The contradictory results pertaining to these taste compounds could relate to differential environments and cultural practices, as well as to possible rootstock–scion interaction.

##### Functional compounds

Pepper fruit carotenoid content, in particular lycopene and β-carotene which is a precursor of vitamin A, can be affected by grafting and is strongly dependent on the choice of rootstock. For instance, red cultivar Fascinato and yellow cultivar Jeanette when grafted onto the rootstock ‘Terrano’ incurred increase in fruit antioxidant capacity and β-carotene content, but not in lycopene content (Chávez-Mendoza et al., 2013). Polyphenols, which constitute a large family of secondary metabolites that act as major antioxidants in the neutralization of free radicals, are abundant in pepper (Colla et al., 2013). Two studies conducted by Spanish researchers showed that grafting effects on the levels of total phenolics in pepper were non-significant (Chávez-Mendoza et al., 2013; López-Marín et al., 2013; Sánchez-Torres et al., 2016).

Pepper fruit contains significant important amounts of ascorbic acid, however, currently available studies have presented conflicting results concerning the variation in vitamin C content in response to grafting (Gisbert et al., 2010; Chávez-Mendoza et al., 2013; López-Marín et al., 2013; Sánchez-Torres et al., 2016). For example, Chávez-Mendoza et al. (2013) observed a significant enhancement of ascorbic acid in grafted pepper plants; but the same effect was not confirmed by López-Marín et al. (2013). The former authors concluded that variation in vitamin C depends on both scion–rootstock combinations and growing conditions, such as plant shading. Nevertheless, other authors reported that grafting had no effect on pepper content in ascorbic acid, such as Gisbert et al. (2010) and Sánchez-Torres et al. (2016) who found no differences when two commercial pepper hybrid cultivars (Almuden and Coyote) were grafted onto two rootstocks (‘Foc’ and Charlot’). In light of the above studies, it might be inferred that high genotypic dependence of this quality trait in pepper scions likely confounds more limited rootstock effects.

## Methodological Approaches and Postulates in Assessing Grafting Effects

### Homeografting vs. Heterografting

In attempting to discern the effects of various rootstocks on the fruit quality of annual crops, an implicit postulate is whether the observed responses stem not entirely from the rootstock but partly from the grafting process itself. In a number of studies, focused mainly on melon, this postulate has been addressed by using homeografts, i.e., self-grafted controls, apart from non-grafted controls. In a study assessing melon transplant growth for 10 days in a hydroponic system, Aloni et al. (2011) found that self-grafting reduced salinity-induced oxidative stress and improved the growth of homeografts compared to both non-grafted control and heterografts on interspecific hybrid TZ148; spanning, however, only a brief vegetative period this could be considered a transient post-transplanting effect. At 30 days after planting, Edelstein et al. (2011) found no difference in shoot and root dry weights between self-grafted and non-grafted treatments of either Galia melon or pumpkin. Additionally, Galia homeografts yielded no differences against non-grafted control in instrumental measurements of quality (SSC and firmness) but only sporadic differences in sensory evaluation, whereas in the case of honeydew melon self-grafting and non-grafting showed no differences in any respect of quality (Guan et al., 2014, 2015); moreover, differences in yield parameters of either scion type were not identified, as was also the case with a wide range of homeografts and non-grafted melon cultivars tested by Schultheis et al. (2015). Notwithstanding the above findings, it cannot be precluded that the grafting process in itself affects plant physiological responses to the growth environment; for instance, improved growth of homeografted melon under salinity stress (Orsini et al., 2013), and improved water relations and xylem water transport efficiency (Agele and Cohen, 2009). Nevertheless, the potential effects of homegrafting seem to pertain chiefly to the early vegetative stages of grafted transplants, as there is no convincing evidence of a lasting effect expressed at the reproductive stage on the quality characteristics of the fruit, which are unequivocally rootstock-mediated. Further to the numerous reports on rootstock mediation of fruit quality discussed in the context of the current review, the effect of heterografting was recently highlighted by high throughput sequencing which revealed that 787 and 3485 genes, associated with primary and secondary metabolism, hormone signaling, transcription factor regulation, transport, and responses to stimuli, were differentially expressed in watermelon when grafted onto bottle gourd and squash rootstocks, respectively, as opposed to self-grafted watermelon (Liu et al., 2016).

### Confounding Harvest Maturity with Rootstock Effects on Quality

Quality is configured in the course of post-anthesis ontogeny and ripening, hence harvesting at optimum maturity is particularly critical for non-climacteric annual fruits (e.g., watermelon, honeydew melon, cucumber, eggplant, and bell pepper), the quality of which is configured while on the plant and steadily deteriorates postharvest at a temperature-dependent rate; whereas the quality of climacteric fruits (e.g., muskmelon and tomato), provided they are harvested physiologically mature, will improve postharvest with the onset of the climacteric and ethylene-induced changes in physicochemical composition (Kader, 1999, 2008). Harvest maturity is a major parameter of quality configuration in annual fruit crops which owed to be standardized before sound conclusions can be drawn on the effects of grafting thereon. Most studies reporting rootstock-mediated effects on fruit quality have relied on an implicit assumption of synchronous ripening behavior in grafted and non-grafted plants, and either did not explicitly monitor harvest maturity or have implicitly relied on crop-specific empirical maturity indices, such as skin color development, formation of abscission layer, or axillary tendril wilting and ground spot formation, which may provide only limited standardization of maturity (Reid, 2002); however, satisfactory standardization must rely principally on the age of the fruit monitored in days post-anthesis (Kyriacou et al., 1996, 2016). The simultaneous harvest of grafted and non-grafted plants is inherently problematic as it overlooks the potential effect of grafting on fruit ripening behavior and may yield misleading results regarding rootstock effects on quality (Davis et al., 2008a). This may partly explain contradictory reports on rootstock-mediated effects on quality and widespread rootstock–scion interaction. The significant effect of vigorous commercial rootstocks, especially of interspecific hybrids, on the yield characteristics of grafted plants indicates that grafting may mediate source–sink relations in the course of ripening. Recent work has demonstrated that grafting watermelon on vigorous rootstocks can increase crop load and retard ripening events responsible for physicochemical changes in fruit composition (Soteriou et al., 2014; Kyriacou et al., 2016). In this case, the apparent effect of grafting on key quality traits, such as the concentration of non-structural carbohydrates and the SSC, was found insignificant and differences between grafted and non-grafted treatments were sourced to the interaction of grafting with maturity due to asynchronous ripening. The synthesis of key pigments responsible for fruit color development, such as lycopene, is also highly dependent on the stage of maturity. Monitoring pigment levels and colourimetric values during the course of fruit ripening has revealed significant grafting × maturity interaction which, in the absence of standardized sampling, might be taken as mere grafting effect (Soteriou et al., 2014). Further complications in interpreting grafting effects might be compiled by recurrent harvests from the same plants and from non-discriminate data analysis on fruits sampled from different orders of fruit clusters.

## Biological Mechanisms Affecting Quality in Grafted Annual Fruit Crops

The interactions between rootstock and scion are highly complex, but increasing investigations in this field have recently shed considerable light on the biological mechanisms involved (Goldschmidt, 2014; Wang et al., 2016). It is widely accepted that metabolic substances could be transferred from one grafting partner to the other, including signaling molecules that may cause large biological effects. Hormonal signaling is implicated in graft union formation, rootstock–scion communication, growth, yield, and potentially flowering and fruit quality (Aloni et al., 2010). Specific studies have documented that grafting also enables long-distance movement of RNA through the phloem (Lucas et al., 2001), the functional importance of which, however, needs to be determined individually. For example, long-distance movement of mutant mRNA from the rootstock to the wild-type tomato scions caused obvious change in leaf morphology, suggesting that translocated RNAs were functional (Kim et al., 2001). Many other phloem-mobile mRNAs have been identified (Harada, 2010), and recent work with grafted grapevines suggests that genomic-scale mRNA exchange across graft junctions is widespread in grafted fruit and vegetable species (Yang et al., 2015). From the different mRNA patterns, it might be concluded that the profile of mobile mRNAs has specific genotype- and environment-dependent characteristics able to modulate plant performance (Yang et al., 2015). But what determines that an mRNA is selected for long-distance movement? Current knowledge is increasing regarding RNA motifs that trigger mobility, the extent of mRNA transport, and the potential for post-transport translation of mRNAs into functional proteins. Long-distance transport of gibberellic acid insensitive-RNA via the phloem altered leaf morphology and raised the question whether RNA delivery may be regulated by sequence motifs conserved between plant families (Haywood et al., 2005). Further studies indicated that coding sequences, 3′ untranslated regions, and also the structure of the RNA might be factors to target for RNA long-distance movement (Huang and Yu, 2009). A recent study exploring the motifs triggering mobility of mRNA demonstrated that tRNA-derived sequences with specific structures are sufficient to mediate mRNA transport and seem necessary for the mobility of a large number of endogenous transcripts that can move through graft junctions (Zhang W.N. et al., 2016). However, it must be considered that the great number of mobile mRNAs identified by combining interspecific grafting with high throughput RNA sequencing, indicate that a postulated tissue-specific gene expression profile might not be predictive for the actual plant body part in which a transcript exerts its function (Thieme et al., 2015).

Furthermore, it also has to be taken into account that grafting itself induces differential gene expression. For example, transcriptomic analysis of grapevine scions demonstrated extensive transcriptional re-programming after heterografting onto two different genotypes (Cookson and Ollat, 2013). While the choice of rootstock genotype had little effect on gene expression in the shoot apex, it was concluded that homeografting and heterografting was the major factor regulating gene expression (Cookson and Ollat, 2013). Besides, heterografting with non-self rootstocks induced genes involved in stress responses at the graft interface when compared with homeografted controls (Cookson et al., 2014). Genome-wide investigation using high-throughput sequencing and comparative analysis of grafting-responsive mRNA in watermelon grafted onto bottle gourd and squash rootstocks identified genes associated with primary and secondary metabolism, hormone signaling, transcription factors, transporters, and response to stimuli, which provide an excellent resource to further elucidate the molecular mechanisms underlying grafting-induced physiological processes (Liu et al., 2016). In addition to protein-encoding mRNAs, various non-coding small RNAs have been shown to move long distances via phloem sap in grafts. Some specifically accumulate in response to nutrient deprivation (Buhtz et al., 2010) with potential signaling role in long distance regulation of gene expression (Pant et al., 2008). Furthermore, it was reported that transgene derived small RNAs from endogenous inverted repeat loci are mobile through the graft union with direct epigenetic modification in recipient cells (Molnar et al., 2010). However, it also has to be considered that grafting itself induces differential expression of microRNAs, as aptly demonstrated by high-throughput sequencing in watermelon grafted onto different rootstocks (Liu et al., 2013). This leads to the suggestion that microRNAs playing an important role in diverse biological and metabolic processes might regulate plant development and adaptation to stress by grafting-induced alterations (Liu et al., 2013).

Despite the mobility of RNA, the transport of various macromolecules through the phloem has received increasing interest following the discovery that FLOWERING LOCUS T protein moves from leaves to the shoot apical meristem where it induces flowering (Corbesier et al., 2007). Paultre et al. (2016) further addressed movement of proteins through the phloem and showed that many proteins in companion cells can get swept away by the translocation stream without resembling a specific protein signal (Paultre et al., 2016). These data reveal that proteins are lost constitutively to the translocation stream, making the identification of unique systemic phloem signals a difficult challenge for the future. However, movement of proteins across graft unions is not restricted to the phloem path as it was demonstrated in transgrafting pathogen resistant, genetically engineered rootstocks with wild type scions. Rootstocks expressing transgenic polygalacturonase inhibiting protein (PGIP) as components of the defense against invasion with pathogens, onto which non-expressing scions were grafted, do not export the respective encoding nucleic acid rather than the PGIP protein itself via the xylem system (Aguero et al., 2005). Furthermore, the PGIP protein in the wild-type scion tissue grafted onto PGIP-expressing genetically engineered rootstocks reduced pathogen damage in scion tissues (Haroldsen et al., 2012). Thus, defense factors in roots can be made available to scions via grafting, improving the vigor, quality, and pathogen resistance of the food-producing scion and its crop (Guan et al., 2012).

It has long been questioned whether grafting might stimulate heritable changes in the scion. Studies have documented that grafting enables the exchanges of DNA molecules between the grafting partners, thus providing a molecular basis for grafting-induced genetic variation (Stegemann and Bock, 2009). By grafting sexually incompatible species, it was further shown that complete chloroplast genomes can travel across the graft junction from one species into another (Stegemann et al., 2012). Additionally, it has been demonstrated that upon grafting entire nuclear genomes can be transferred between plant cells (Fuentes et al., 2014). Although these alterations are localized to the contact zone between scion and rootstock it indicates that the changes may become heritable via lateral shoot formation from the graft site. Hence, it demonstrates that large DNA pieces or entire plastid genomes can travel into the scion as a prerequisite of graft hybridization (Liu et al., 2010). Heritable changes in the scion might also be the result of epigenetic effects associated with grafting. Wu and co-workers demonstrated that in solanaceous plants heterografting causes extensive alteration of DNA methylation patterns in a locus-specific manner, especially in the scions (Wu et al., 2013). They further detected that altered methylation patterns could be inherited to sexual progenies with some sites showing additional alterations or revisions. Such putatively heritable changes in the DNA methylation pattern of solanaceous scion genomes were extended to the Cucurbitaceae. Using methylation-sensitive amplified polymorphism markers, global DNA methylation changes in scions of cucumber, melon and watermelon heterografted onto pumpkin rootstocks were observed (Avramidou et al., 2015). The differential epigenetic marking in different rootstock–scion combinations will enable the development of epi-molecular markers for generation and selection of superior quality grafted vegetables in the future (Avramidou et al., 2015).

## Concluding Remarks and the Challenges Ahead

Regarded primarily as a phytoprotective measure and as a means to alleviate abiotic plant stress, the grafting of annual fruit crops carries significant, crop-specific implications for fruit quality and nutritive value. The positive effects of vigorous interspecific rootstocks on scion performance are often reflected on fruit size, particularly in crops such as watermelon, cucumber, and tomato, whereas fruit shape constitutes a trait predominantly governed by the scion genotype. Similarly, grafting effect on exocarp and mesocarp thickness is limited and inferior to that of the scion genotype, moreover it interacts with fruit maturity. Variation in the epidermal and pulp colouration of annual fruits, determined by changes in pigment concentrations, can be influenced by grafting directly and indirectly through its interaction with fruit ripening behavior; such an interaction is common for watermelon while colouration effects on tomato, melon and pepper appear strongly rootstock-specific.

Fruit texture can be highly affected by grafting as manifested most consistently in the case of watermelon grafted on interspecific cucurbit rootstocks which generally increase pulp firmness; whereas loss of firmness in melon can reflect latent rootstock–scion incompatibility. Arguably the most important sensorial attribute is fruit sweetness, elicited by soluble carbohydrates whose concentration is liable to the effects of grafting. Rootstock-mediated changes in sweetness may also encompass changes in melon starch content and in the relative proportions of hexoses to sucrose. Decrease in sugars is not an infrequent response to grafting, but the increments of reported decrease are in general not highly critical for overall quality and marketability. Nevertheless, additional work is warranted across fruit crops to elucidate widespread rootstock–scion interactions regarding sugar content. While advances have been made with regards to grafting effects on fruit aroma profile and the levels of secondary bioactive phytochemicals, these areas remain largely uncharted, underscored by conflicting reports and warranting further research before grafting may constitute a reliable tool for improving fruit sensorial and nutritional quality.

Disparate results on critical quality attributes such as sugar content and aroma profile often reflect a wider effect of grafting on flowering behavior and post-anthesis ripening events partly mediated by changes in crop load. Further complications can be compounded by sampling practices such as recurrent harvests from the same plants and non-standardization of harvest maturity. From a physiological standpoint, the grafting process in itself may modulate plant responses to the growth environment, but these effects of homeografting appear concerted mainly in the early vegetative stages following graft union formation; unlike heterografting whose effects may pervade the reproductive stage configuring fruit quality characteristics. Hormonal signaling, however, is implicated in graft union formation, rootstock–scion communication, growth, yield, and potentially flowering and fruit quality. Moreover, the long-distance phloem transport of genomic-scale mRNA across graft unions is widespread in grafted fruit and vegetable species. Yet, additional knowledge is required on RNA motifs that trigger mobility, the extent of mRNA transport, and the potential for its post-transport translation into functional and tissue-specific proteins. The identification of systemic phloem signals, including noncoding microRNAs and proteins with diverse roles in post-grafting biological and metabolic processes, will prove valuable in understanding grafting effects on fruit quality. Ultimately, the identification of inheritable locus-specific alterations in scion DNA methylation patterns may enable the development of epi-molecular markers for generation and selection of superior quality grafted vegetables in the future.

## Author Contributions

The review invitation was addressed to DS. Authors’ contributed as follows: Introduction and conclusion – all authors; Watermelon and melon – MK; Cucumber, eggplant, and pepper – YR and GC; Tomato – DS; Methodological approaches and postulates – MK; and Biological mechanisms affecting quality – RZ.

## Conflict of Interest Statement

The authors declare that the research was conducted in the absence of any commercial or financial relationships that could be construed as a potential conflict of interest.
